# Identification of potential biomarkers and therapeutic targets related to post-traumatic stress disorder due to traumatic brain injury

**DOI:** 10.1186/s40001-024-01640-x

**Published:** 2024-01-11

**Authors:** Peng Qi, Mengjie Huang, Xuewen Ren, Yongzhi Zhai, Chen Qiu, Haiyan Zhu

**Affiliations:** 1 Department of Emergency, First Medical Center of Chinese, PLA General Hospital, 28 Fuxing Road, Beijing, 100853 China; 2 Department of Nephrology, First Medical Center of Chinese, PLA General Hospital, 28 Fuxing Road, Beijing, 100853 China; 3 Department of Orthopedics, Fourth Medical Center of Chinese, PLA General Hospital, Beijing, 100853 China

**Keywords:** Traumatic brain injury, Post-traumatic stress disorder, Bioinformatic analysis, Differentially expressed genes, Biomarker

## Abstract

**Background:**

Post-traumatic stress disorder (PTSD), a disease state that has an unclear pathogenesis, imposes a substantial burden on individuals and society. Traumatic brain injury (TBI) is one of the most significant triggers of PTSD. Identifying biomarkers associated with TBI-related PTSD will help researchers to uncover the underlying mechanism that drives disease development. Furthermore, it remains to be confirmed whether different types of traumas share a common mechanism of action.

**Methods:**

For this study, we screened the eligible data sets from the Gene Expression Omnibus (GEO) database, obtained differentially expressed genes (DEGs) through analysis, conducted functional enrichment analysis on the DEGs in order to understand their molecular mechanisms, constructed a PPI network, used various algorithms to obtain hub genes, and finally evaluated, validated, and analyzed the diagnostic performance of the hub genes.

**Results:**

A total of 430 upregulated and 992 down-regulated differentially expressed genes were extracted from the TBI data set. A total of 1919 upregulated and 851 down-regulated differentially expressed genes were extracted from the PTSD data set. Functional enrichment analysis revealed that the differentially expressed genes had biological functions linked to molecular regulation, cell signaling transduction, cell metabolic regulation, and immune response. After constructing a PPI network and introducing algorithm analysis, the upregulated hub genes were identified as VNN1, SERPINB2, and ETFDH, and the down-regulated hub genes were identified as FLT3LG, DYRK1A, DCN, and FKBP8. In addition, by comparing the data with patients with other types of trauma, it was revealed that PTSD showed different molecular processes that are under the influence of different trauma characteristics and responses.

**Conclusions:**

By exploring the role of different types of traumas during the pathogenesis of PTSD, its possible molecular mechanisms have been revealed, providing vital information for understanding the complex pathways associated with TBI-related PTSD. The data in this study has important implications for the design and development of new diagnostic and therapeutic methods needed to treat and manage PTSD.

## Introduction

According to statistics, more than 50 million people worldwide suffer from traumatic brain injury (TBI) each year, and over the past 30 years, both the incidence and prevalence have increased [1, 2]. This type of injury imposes a significant burden on global healthcare institutions. In addition, recent studies have found that TBI will evolve over time [3]. Over the past decade, there has been a fundamental shift in understanding TBI, as experts now recognize it as a chronic condition affecting multiple bodily systems [4]. Patients often experience persistent cognitive, emotional, and functional impairments in their daily lives, greatly impacting their quality of life [5]. Among these impairments, post-traumatic stress disorder (PTSD) is one of the most typical presentations [6, 7]. According to the fifth edition of the diagnostic and statistical manual of mental disorders [8], PTSD encompasses four symptomatic clusters. These include the presence of intrusive memories related to the stressful event, persistent avoidance of stimuli associated with the event, negative alterations in mood or cognition, and hyperarousal or excessive vigilance. These symptoms must present within one month following the experience of a distressing or traumatic event. However, symptoms may have a delayed onset in some cases, occurring years later [9]. It is estimated that PTSD affects approximately 5–10% of the global population, with a gender ratio of approximately 1:2, favoring females over males [10]. In countries and regions with an internal conflict backdrop, the prevalence of PTSD is estimated to be even higher, potentially affecting more than 25% of the population [11]. PTSD is considered a heterogeneous disorder, with multiple pathological pathways believed to be involved in its onset and progression. In recent years, various studies have been exploring the possible biological mechanisms that underlie PTSD. These include the hypothalamic–pituitary–adrenal (HPA) axis, neurochemical factors, autonomic nervous system, inflammation, and immune dysregulation, which have all been proposed as potential neuroimmune biomarkers for PTSD [12–16]. However, recent literature review analyses have shown conflicting data on the role of some biomarkers in individuals with PTSD [17]. One of the reasons for such discrepancies stems from current molecular mechanism research concerning PTSD, which relies primarily on animal experiments that necessitate further investigation using human samples [18]. Conversely, the development of the disorder also depends on the characteristics of the trauma and individual risk factors [19]. To screen and explore potential biomarkers as well as the therapeutic targets used to treat PTSD that TBI causes, we conducted further analysis of the molecular mechanisms of TBI-associated PTSD. We utilized publicly available human specimen data sets related to TBI and PTSD and identified differentially expressed genes (DEGs) using bioinformatics methods. Subsequently, we performed GO and KEGG analyses and constructed protein–protein interaction (PPI) networks in order to elucidate the functional classification and the metabolic pathways of the DEGs. We also identified potential hub genes. To investigate whether different traumatic features can influence the molecular mechanisms of PTSD, we introduced a third human specimen data set related to fracture injury for validation. This allowed us to explore whether different traumatic features have shared mechanisms in their impact on PTSD, as well as to determine whether TBI-related PTSD has distinct characteristics that warrant further independent research.

## Methods

### Data set information

The keywords “trauma” and “PTSD” were used as screening terms to search the GEO database. The search criteria were further limited to “Homo sapiens” and “Expression profiling by array.” The search results were analyzed individually, and the original data set literature that met the research criteria was carefully read and reviewed. Finally, the GSE223245 and GSE81761 data sets were identified as subjects for further research. GSE223245 includes 12 TBI patients and 4 healthy controls, while GSE81761 includes 39 PTSD patients and 27 healthy controls. Both data sets collected venous blood for gene detection. The raw data of both data sets were downloaded using R software (version 4.2.1, http://r-project.org/). Then, the “affy” package [20] within the R software was used to read the raw data of the two data sets used for background correction and data normalization.

### Differential gene expression analysis

The “limma” package [21] was used to analyze the two data sets separately. The disease group and control group within each data set were labeled. Differentially expressed genes between the two groups in each data set were obtained by performing comparative analysis. Since the molecular mechanisms of PTSD are not yet well understood, in order to screen for more valuable genes, the criteria were set as a |log2 (fold-change)|> 0.2 and a *p* value < 0.05 to include genes as differentially expressed genes. Those that met these two criteria were considered to have significant differences. Among the selected DEGs, genes with logFC > 0.2 were considered upregulated, while genes with logFC < − 0.2 were considered downregulated.

### GO and KEGG pathway enrichment analysis

The GO database is a database established by the Gene Ontology Consortium and is primarily divided into three semantic categories: cellular component (CC), molecular function (MF), and biological process (BP). GO enrichment analysis can be used to determine the functions of genes and provide preliminary annotations by analyzing the sequence information of the genes [22]. The KEGG database is a database for systematic analysis of gene products and their metabolic pathways in cells and is commonly used for metabolic pathway analysis [23]. The “clusterProfiler” package in R software [24] is used for enrichment analysis of DEGs, followed by the use of the “org.Hs.eg.db” package for ID conversion [25]. Finally, the “ggplot2” package is used to visualize the enrichment analysis results.

### PPI network construction

Analysis of the common differentially expressed genes obtained from the two data sets is uploaded to the STRING database [26] (http://string-db.org/). The STRING database is an online database for searching known protein–protein interaction relationships, which helps in the exploration of core regulatory genes. The output results are imported into the Cytoscape software [27]. Algorithms are used to explore the potential correlations among these common differentially expressed genes.

### Hub gene selection

Hub genes play essential roles in biological processes and often act as key regulators of other genes in different molecular pathways. The Cyto-Hubba plugin [28] in the Cytoscape software can identify hub genes within the PPI network. To avoid selection bias caused by the different algorithms, the hub genes identified by the different algorithms intersect in order to define the final hub genes.

### Preliminary analysis of hub genes

The PANTHER Classification System [29] (http://pantherdb.org/) can be used to classify proteins (and their genes). It is a comprehensive and annotated gene family system database. The Human Protein Atlas [30] (https://www.proteinatlas.org/) is a public database for querying gene expression profiles in different human organs. It can provide a list of the basic RNA and protein expression levels of specific genes. Selecting the “brain” section of the database, we compared the transcriptome data of 13 major brain regions to classify human brain regions and display the standardized RNA expression levels of the 13 brain regions. We can conduct a preliminary analysis of the hub genes using these two commonly utilized public databases.

### Diagnostic performance of hub genes

ROC curve analysis was conducted on each hub gene data set to assess the diagnostic performance of hub genes. The area under the ROC curve (AUC) of the hub genes was calculated using the “pROC” package in R software. This analysis evaluated the accuracy of diagnosis and the ability to recognize diseases.

### Validation of hub genes

PTSD is a heterogeneous disease with multiple pathophysiological pathways potentially involved in its onset and progression. It requires expanding the data set of the different types of trauma to assess whether different events trigger distinct pathophysiological responses [17]. A recent prospective study also indicated a significantly higher proportion of PTSD occurrence after a TBI compared to other injuries, such as fractures [31]. Therefore, in order to differentiate the molecular mechanisms that underlie the impact of different trauma characteristics on PTSD, we introduced a third human specimen data set focusing on fracture injuries for validation. This validation aims to explore whether different trauma characteristics have common mechanisms affecting PTSD and to confirm if the specificity of PTSD related to TBI warrants further independent research.

## Results

### Differential expression gene screening

**Fig. 1:**
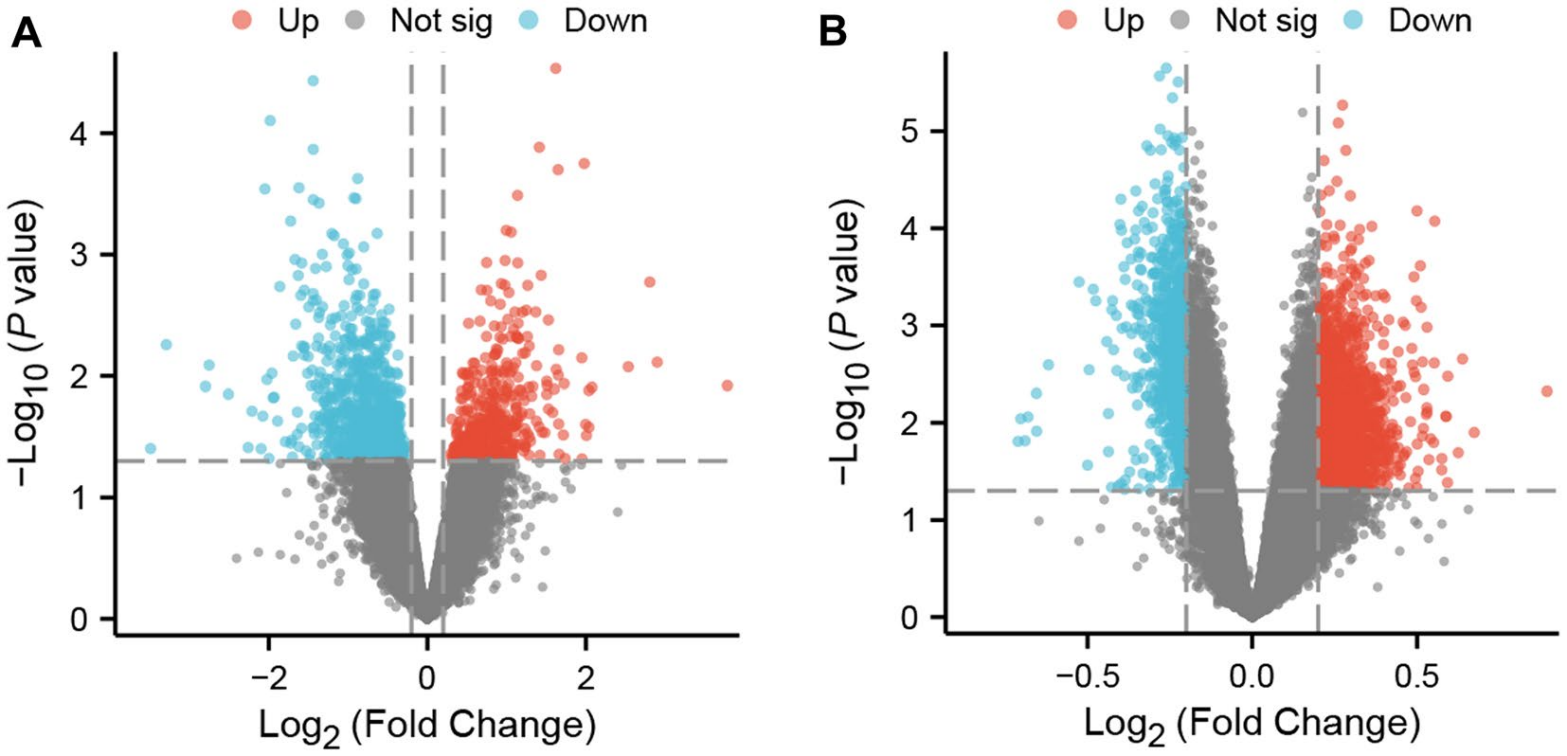
DEGs in the two data sets. **A** Volcano plot of GSE223245; red represents upregulated genes, blue represents downregulated genes, and gray represents nonsignificantly expressed genes. **B** Volcano plot of GSE81761. The criteria for statistically significant differences in DEGs were adjusted |log2(FC)|> 0.2 and p.adj < 0.05 in expression

After data processing of the two data sets, in the GSE223245 TBI data set, there was a total of 430 genes identified as upregulated DEGs that met the criteria of the log2(fold-change) > 0.2 and a *p* value < 0.05. In addition, 992 genes were identified as down-regulated DEGs that met the log2 (fold-change) criteria < 0.2 and a *p* value < 0.05. In the GSE81761 PTSD data set, there were a total of 1919 genes identified as upregulated DEGs that met the criteria of the log2 (fold-change) > 0.2 and a *p* value < 0.05 and 851 genes identified as down-regulated DEGs that met the criteria of the log2 (fold-change) < 0.2 and a *p* value < 0.05. The volcano plot was used to visualize the screening results of the differential expression genes (Fig. 1).

### GO and KEGG pathway enrichment analysis

**Fig. 2:**
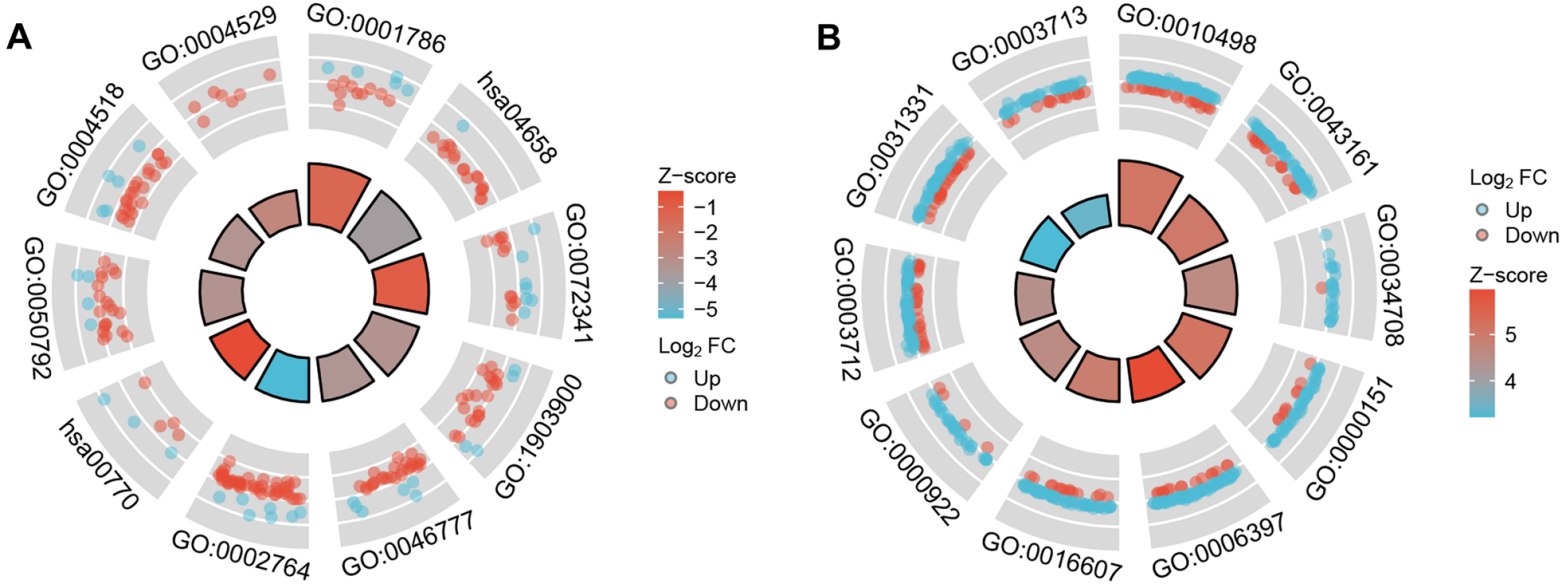
GO terms and KEGG pathway enrichment. **A** Details of GO terms and KEGG pathway enrichment in GSE223245. The inner ring is a bar plot where the height of the bar indicates the significance of the term (− log10 *p* value), and color corresponds to the z score. The outer ring displays scatterplots of the expression levels (logFC) for the genes in each term. **B** Details of GO terms and KEGG pathway enrichment in GSE81761

The “clusterProfiler” package was used to perform GO and KEGG pathway enrichment analysis on the TBI and PTSD data sets independently. The results showed that in the GSE223245 TBI data set, the DEGs were primarily enriched in biological processes (BP) such as viral life cycle regulation (GO:1903900), protein autophosphorylation (GO:0046777), the immune response-regulating signaling pathway (GO:0002764), viral process regulation (GO:0050792), and peptidyl-tyrosine phosphorylation (GO:0018108). In terms of molecular functions (MF), the DEGs were primarily associated with phosphatidylserine binding (GO:0001786), modified amino acid binding (GO:0072341), nuclease activity (GO:0004518), exodeoxyribonuclease activity (GO:0004529), and exodeoxyribonuclease activity that produces 5’-phosphomonoesters (GO:0016895). The enriched KEGG pathways were related to Th1 and Th2 cell differentiation (hsa04658) as well as pantothenate and CoA biosynthesis (hsa00770). In the GSE81761 PTSD data set, the DEGs were predominantly enriched in BP such as proteasomal protein catabolic processes (GO:0010498), proteasome-mediated ubiquitin-dependent protein catabolic processes (GO:0043161), mRNA processing (GO:0006397), positive regulation of cellular catabolic processes (GO:0031331), and the regulation of cellular protein catabolic processes (GO:1903362). In terms of cellular components (CC), the focus was on methyltransferase complexes (GO:0034708), ubiquitin ligase complexes (GO:0000151), nuclear speck (GO:0016607), spindle pole (GO:0000922), and nuclear pore nuclear baskets (GO:0044615). MF was associated with transcription coregulator activity (GO:0003712) and transcription coactivator activity (GO:0003713). The enrichment analysis results indicate that the BPs, CCs, and MFs are associated with different biological pathways, including molecular regulation, cell signaling transduction, cell metabolic regulation, and immune response, suggesting further refinement and classification to facilitate more in-depth research. Visualization was performed using circular plots (Fig. 2) to present the enrichment results clearly. The enrichment results of all the data sets were listed in a ternary table format (Tables 1, 2).

**Table 1:** Details of GO terms and KEGG pathway enrichment in GSE223245

Ontology	ID	Description	GeneRatio	BgRatio	*p* value	p. adjust	z score
BP	GO:1903900	Regulation of viral life cycle	24/1132	139/18800	2.72e-06	0.0145	− 3.266
BP	GO:0046777	Protein autophosphorylation	31/1132	224/18800	1.28e-05	0.0290	− 3.4125
BP	GO:0002764	Immune response-regulating signaling pathway	53/1132	482/18800	1.64e-05	0.0290	− 5.3571
BP	GO:0050792	Regulation of viral process	24/1132	159/18800	2.86e-05	0.0355	− 3.266
BP	GO:0018108	Peptidyl-tyrosine phosphorylation	43/1132	373/18800	3.34e-05	0.0355	− 3.2025
MF	GO:0001786	Phosphatidylserine binding	15/1161	60/18410	3.28e-06	0.0031	− 1.291
MF	GO:0072341	Modified amino acid binding	18/1161	93/18410	1.74e-05	0.0083	− 0.94281
MF	GO:0004518	Nuclease activity	27/1161	204/18410	0.0002	0.0668	− 3.2717
MF	GO:0004529	Exodeoxyribonuclease activity	7/1161	24/18410	0.0005	0.0997	− 2.6458
MF	GO:0016895	Exodeoxyribonuclease activity, producing 5ʹ-phosphomonoesters	7/1161	24/18410	0.0005	0.0997	− 2.6458
KEGG	hsa04658	Th1 and Th2 cell differentiation	18/522	92/8164	1.65e-05	0.0052	− 3.7712
KEGG	hsa00770	Pantothenate and CoA biosynthesis	7/522	21/8164	0.0002	0.0353	− 0.37796

**Table 2:** Details of GO terms and KEGG pathway enrichment in GSE81761

Ontology	ID	Description	GeneRatio	BgRatio	*p* value	p. adjust	z score
BP	GO:0010498	Proteasomal protein catabolic process	91/1964	496/18800	5.79e-08	0.0003	5.1366
BP	GO:0043161	Proteasome-mediated ubiquitin-dependent protein catabolic process	76/1964	414/18800	6.94e-07	0.0020	5.0471
BP	GO:0006397	mRNA processing	85/1964	500/18800	4.08e-06	0.0080	5.9656
BP	GO:0031331	Positive regulation of cellular catabolic process	76/1964	449/18800	1.52e-05	0.0224	3.2118
BP	GO:1903362	Regulation of cellular protein catabolic process	48/1964	258/18800	5.26e-05	0.0619	2.0207
CC	GO:0034708	Methyltransferase complex	25/2047	92/19594	5.38e-06	0.0025	4.6
CC	GO:0000151	Ubiquitin ligase complex	57/2047	302/19594	7.09e-06	0.0025	5.1657
CC	GO:0016607	Nuclear speck	69/2047	411/19594	4.85e-05	0.0095	4.9358
CC	GO:0000922	Spindle pole	35/2047	169/19594	5.81e-05	0.0095	4.5638
CC	GO:0044615	Nuclear pore nuclear basket	7/2047	12/19594	6.62e-05	0.0095	1.8898
MF	GO:0003712	Transcription coregulator activity	85/2017	497/18410	1.96e-05	0.0216	4.4471
MF	GO:0003713	Transcription coactivator activity	50/2017	266/18410	9.47e-05	0.0522	3.3941

### PPI network construction

**Fig. 3:**
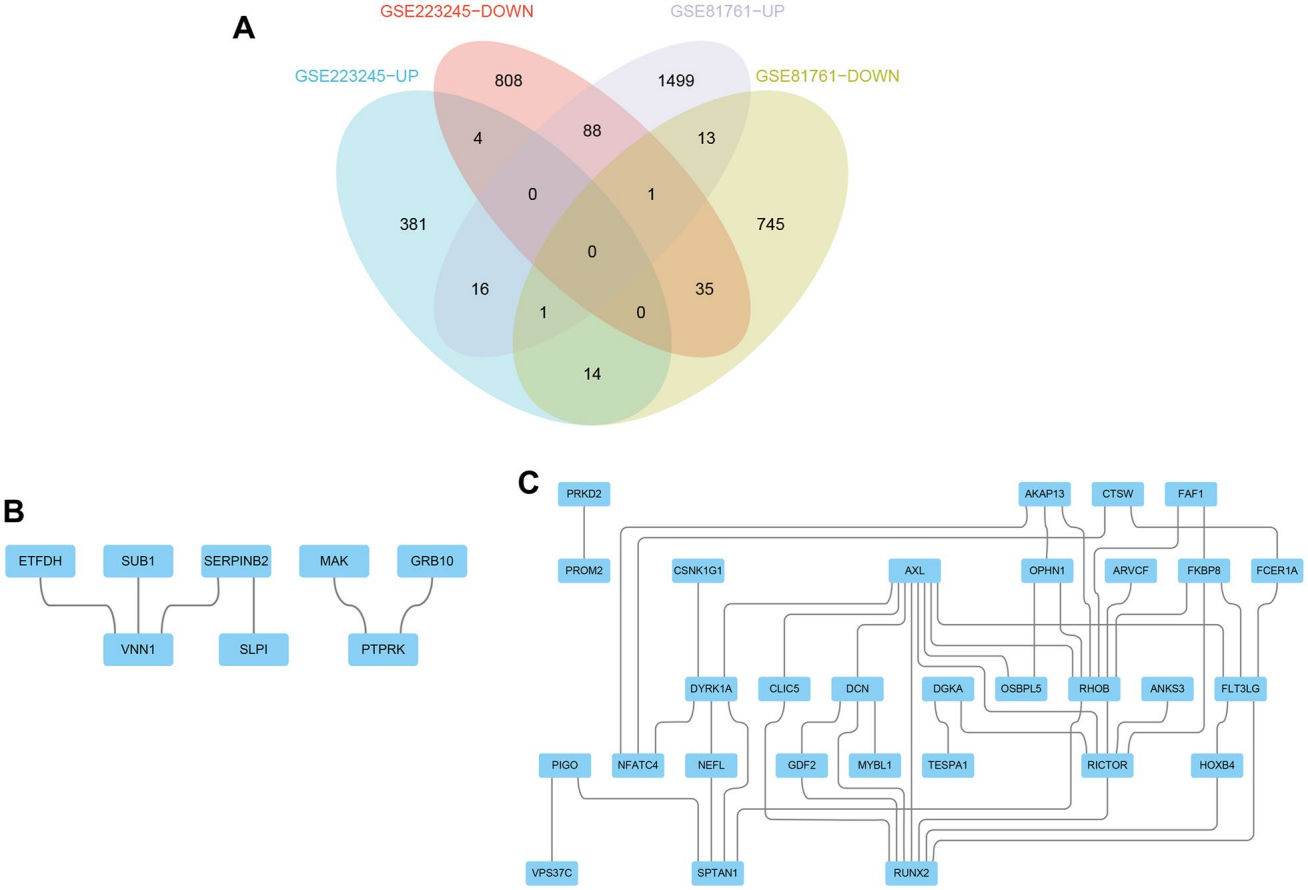
Venn diagram and PPI network. **A** The Venn diagram shows the co-DEGs of the two data sets. **B** A PPI network was constructed using coupregulated genes. **C** A PPI network was constructed using codownregulated genes

The overlapping upregulated and downregulated DEGs from the two data sets were analyzed separately using the “VennDiagram” package (Fig. 3A). This resulted in 17 commonly upregulated DEGs and 36 commonly downregulated DEGs. These genes were uploaded to the STRING database to construct the PPI network. The generated data was then exported and imported into the Cytoscape software to visualize and analyze the PPI network, resulting in a graphical representation of PPI (Fig. 3B, C).

### Hub gene selection

**Fig. 4:**
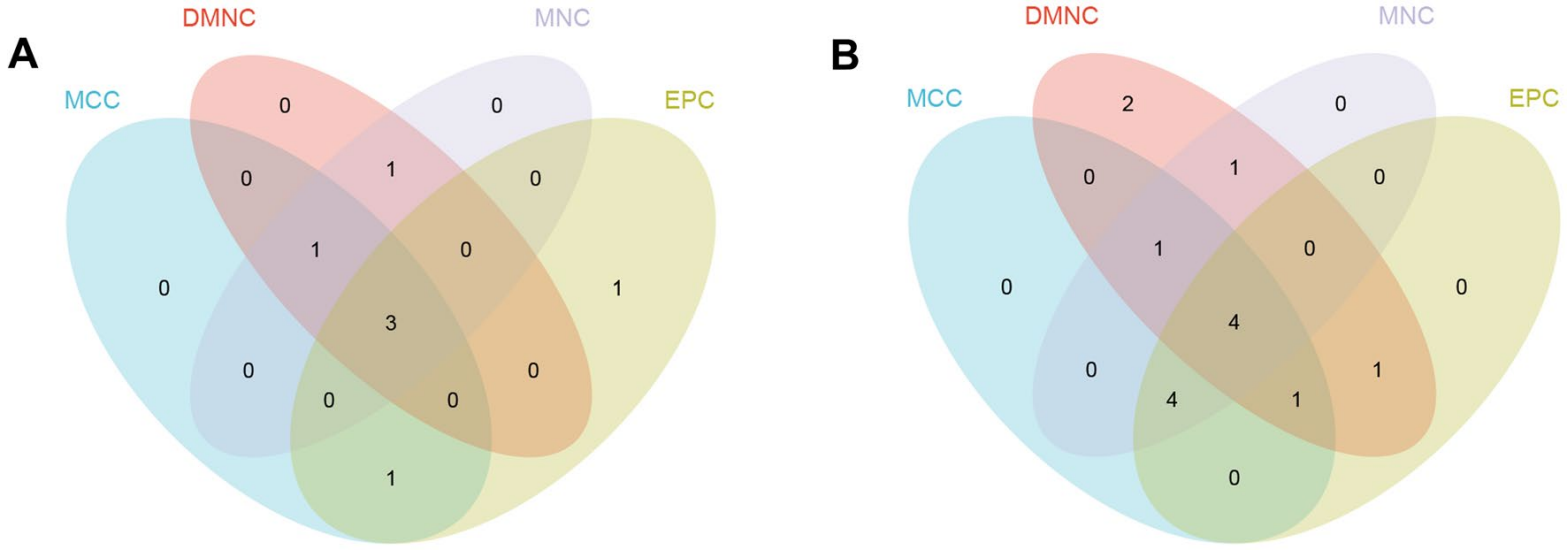
The hub genes were merged by the MCC, DMNC, MNC, and EPC algorithms. **A** VNN1, SERPINB2 and ETFDH were upregulated hub genes. **B** FLT3LG, DYRK1A, DCN and FKBP8 were downregulated hub genes

The Cyto-Hubba plugin in the Cytoscape software is a widely used network visualization tool that allows for the discovery of key target information for various types of biological data, such as gene regulation and signal transduction. The plugin assigns a value to each gene using topological network algorithms and ranks them to identify the hub genes. In order to avoid bias caused by different topological analysis methods, four methods were used for analysis: MCC, DMNC, MNC, and EPC. The results were then merged and analyzed using the “VennDiagram” package, resulting in the identification of the hub genes (Fig. 4). The upregulated hub genes identified were VNN1, SERPINB2, and ETFDH, while the downregulated hub genes identified were FLT3LG, DYRK1A, DCN, and FKBP8.

### Preliminary analysis of hub genes

**Fig. 5:**
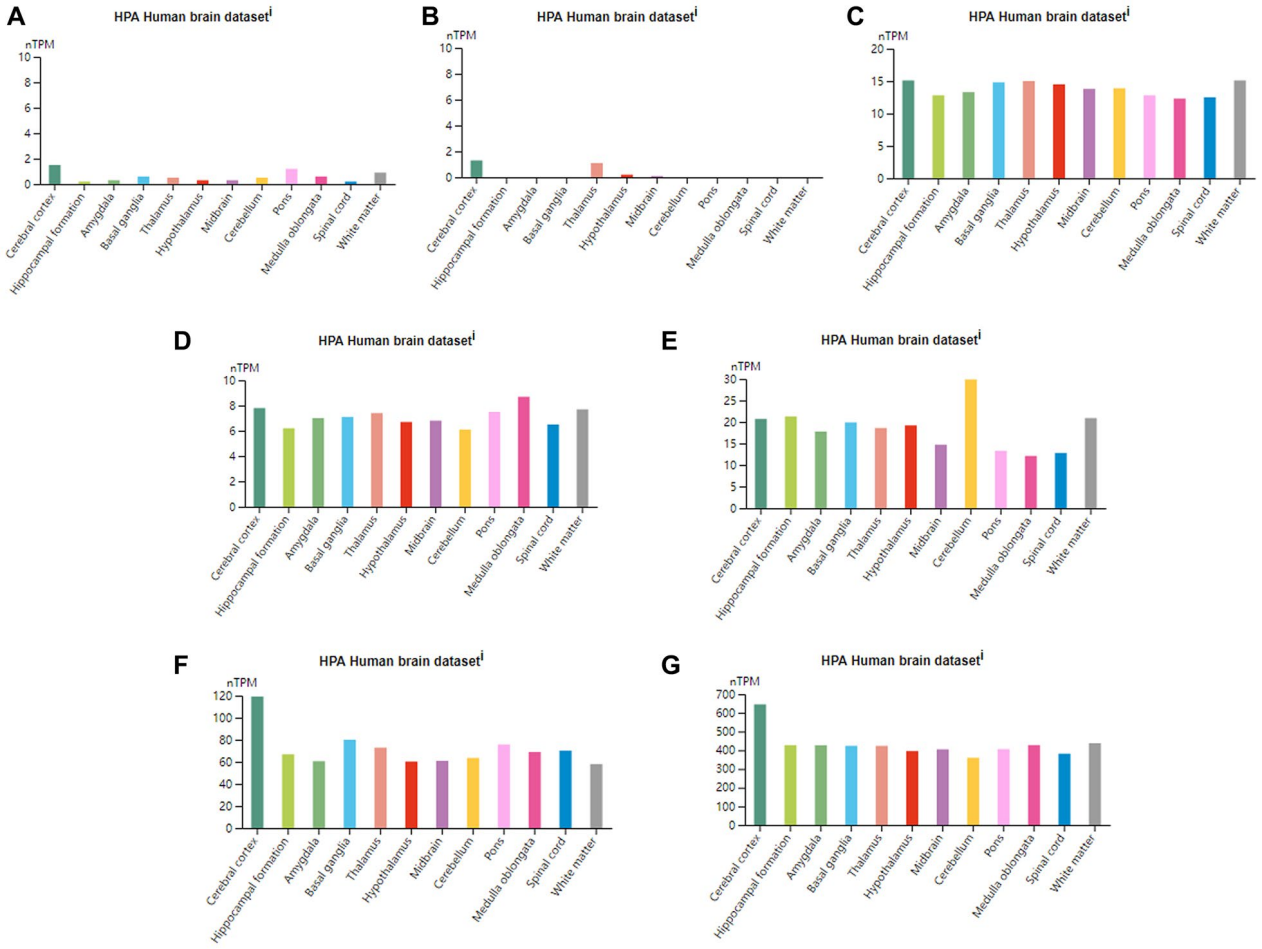
The Human Protein Atlas was used to analyze the hub genes. Regional classification in the human brain by comparing transcriptome data aggregated to 13 major regions of the brain. Normalized RNA expression levels shown for the 13 brain regions. Color coding is based on brain region, and the bar shows the highest expression among the subregions included. **A** VNN1 **B** SERPINB2 **C** ETFDH **D** FLT3LG **E** DYRK1A **F** DCN **G** FKBP8

First, the hub genes were subjected to a one-stop annotation analysis using the PANTHER Classification System (http://pantherdb.org/) to obtain an initial understanding of their functions (Table 3). Considering that the hub genes may exhibit differential expression in different brain regions during cranial brain injury processing, the Human Protein Atlas database (https://www.proteinatlas.org/) was used to compile the basic RNA and protein expression levels of the hub genes. These expression levels were then displayed across different brain regions based on standardized RNA expression levels. The color coding was based on the brain regions, and the bar chart depicts the highest expression levels within the included subregions (Fig. 5).

**Table 3:** The PANTHER classification system of hub genes

Gene	Gene name	Gene ID	PANTHER Family/Subfamily	PANTHER protein class
VNN1	Pantetheinase	HUMAN|HGNC = 12,705|UniProtKB = O95497	PANTETHEINASE (PTHR10609:SF16)	Hydrolase
SERPINB2	Plasminogen activator inhibitor 2	HUMAN|HGNC = 8584|UniProtKB = P05120	PLASMINOGEN ACTIVATOR INHIBITOR 2 (PTHR11461:SF61)	Protease inhibitor
ETFDH	Electron transfer flavoprotein–ubiquinone oxidoreductase, mitochondrial	HUMAN|HGNC = 3483|UniProtKB = Q16134	ELECTRON TRANSFER FLAVOPROTEIN-UBIQUINONE OXIDOREDUCTASE, MITOCHONDRIAL (PTHR10617:SF107)	Oxidoreductase
FLT3LG	Fms-related tyrosine kinase 3 ligand	HUMAN|HGNC = 3766|UniProtKB = P49771	FMS-RELATED TYROSINE KINASE 3 LIGAND (PTHR11032:SF1)	Cytokine
DYRK1A	Dual specificity tyrosine–phosphorylation-regulated kinase 1A	HUMAN|HGNC = 3091|UniProtKB = Q13627	DUAL SPECIFICITY TYROSINE-PHOSPHORYLATION-REGULATED KINASE 1A (PTHR24058:SF121)	Protein modifying enzyme
DCN	Decorin	HUMAN|HGNC = 2705|UniProtKB = P07585	DECORIN (PTHR45712:SF14)	–
FKBP8	Peptidyl-prolyl cis–trans isomerase FKBP8	HUMAN|HGNC = 3724|UniProtKB = Q14318	PEPTIDYL-PROLYL CIS–TRANS ISOMERASE FKBP8 (PTHR46512:SF3)	Chaperone

### Diagnostic performance of hub genes

**Fig. 6:**
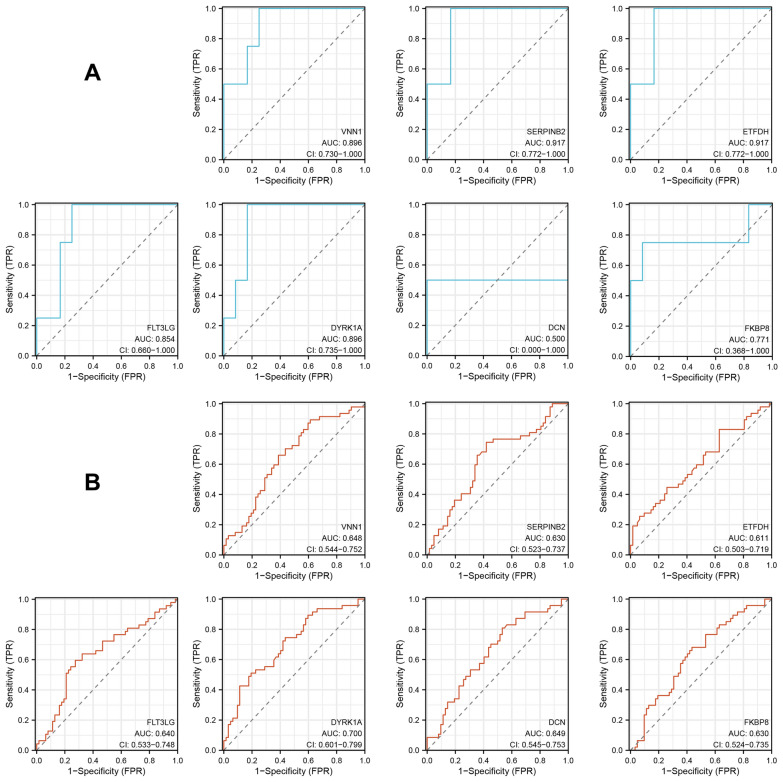
GEO data set was used to verify the diagnostic performance of the hub genes. **A** ROC curve of hub genes in the GSE223245 data set. **B** ROC curve of hub genes in the GSE81761 data set

To validate the diagnostic performance of the hub genes, we conducted a ROC curve analysis based on the two GEO data sets (Fig. 6). The results showed that, except for DCN, all of the hub genes exhibited good diagnostic performance within the TBI data set. Considering that the gene expression differences between the PTSD patients and the normal controls may be relatively small, thus, the diagnostic performance of the hub genes within the PTSD data set may not be as expected. However, the results indicated that the area under the ROC curve for all of the hub genes in the PTSD data set was > 60%. Therefore, we believe that the selected hub genes from this study are key genes in the pathogenesis of TBI-related PTSD and possess a significantly high diagnostic value.

### Validation of hub genes

To speculate whether different trauma characteristics affect the molecular mechanisms of PTSD, we introduced a third data set for validation due to the involvement of different biological pathways containing different trauma characteristics[19]. Using the same selection criteria as before, we selected the GSE93138 fracture data set as the validation data set. After preprocessing the raw data, screening DEGs, constructing PPI networks, and identifying hub genes, we obtained the upregulated hub genes in the fracture and PTSD data sets: RBX1, COMMD8, COMMD6, DCUN1D1, DCUN1D5, TCEB1, COPS2, and CUL3. The downregulated hub genes identified were CD79B, CD79A, CD40, PAX5, FCER2, HNRNPH1, G3BP1, IL2RG, SRRM2, and APP. By comparing the hub genes between the different groups, it was observed that for PTSD, the target genes involved in the various trauma mechanisms are not identical. Thus, it can be reasonably inferred that there are differences in the molecular mechanisms during the occurrence and development of PTSD under different trauma characteristics. This also explains why some biomarkers show contradictory results in PTSD patients [17]. These validation results suggest that researchers should pay attention to the effects of the trauma characteristics when studying the mechanisms of PTSD, thus providing an increase in best practices used in guiding clinical practice and research.

## Discussion

PTSD is a chronic mental disorder that leads to a decrease in patient quality of life and an increase in economic burden. Exposure to traumatic stressors is a triggering factor in the development of PTSD [32]. The first-line intervention for patients with PTSD is psychological therapy, either trauma-focused or non-trauma-focused [18]. However, these psychological therapies share common issues, such as prolonged treatment duration and significant individual variation. Many patients are unable to eliminate PTSD symptoms even after receiving treatment, leading to poor adherence to long-term therapy [33]. Currently, there are no specific drugs that have been proven to be effective in treating PTSD [34]. The two first-line medications approved by the US FDA for the clinical treatment of PTSD are sertraline and paroxetine; both are selective serotonin reuptake inhibitors (SSRIs), which are commonly used as antidepressants [35]. However, these medications have issues such as low efficacy rates, delayed onset of action, and significant adverse effects [36]. Therefore, conducting in-depth research on the pathogenesis of PTSD, identifying new potential treatment targets, and exploring novel treatment strategies are of significant importance in improving the treatment outcomes for PTSD patients. In recent years, there has been increased attention surrounding PTSD biomarker research [37]. These biomarkers may aid in screening and supporting early detection of PTSD, as well as identifying drug targets that will lead to timely intervention and better outcomes for individuals suffering from PTSD [38–40]. Based on our research findings, we have identified VNN1, SERPINB2, and ETFDH as upregulated hub genes and FLT3LG, DYRK1A, DCN, and FKBP8 as downregulated hub genes associated with PTSD following TBI. According to the review of previous literature, Vanin-1 (VNN1) is a glycosylphosphatidylinositol-anchored pantetheinase that catalyzes the hydrolysis of pantetheine into cysteamine and pantothenic acid [41]. Studies have shown that VNN1 plays a role in oxidative stress, inflammation, and cell migration [42–45]. In addition, VNN1’s cysteamine pantetheinase activity contributes to local tissue adaptation, injury resilience, and tissue stress tolerance [46]. A recent study demonstrated that gene knockout of VNN1 prevented organ damage from progressing from acute to chronic stages [47]. These mechanistic studies and animal experiments provide theoretical support for the role of VNN1 in PTSD. Serpin family B member 2 (SERPINB2), also known as plasminogen activator inhibitor-2 (PAI-2), is an inhibitor of the extracellular protease plasminogen activator and is expressed in various cell types [48]. SERPINB2 participates in multiple cellular functions, including cell survival, cell differentiation, inflammation, immunity, cell adhesion, migration, and extracellular matrix remodeling in multiple disease states via its interactions with intra and extracellular proteins [49–51]. Despite extensive research on SERPINB2, no consensus exists on its pathophysiological functions [52]. However, a recent study suggests that the downregulation of SERPINB2 may have potential therapeutic value in relieving chronic visceral pain syndromes, such as chronic pelvic pain syndrome within the urological system [53]. This improvement in chronic pain-related conditions also suggests that SERPINB2 may hold some value in the treatment of PTSD. Electron transfer flavoprotein dehydrogenase (ETFDH), also known as ETF–ubiquinone oxidoreductase (ETF–QO), is a protein located in the inner mitochondrial membrane and plays a central role in the electron transfer system [54]. It is closely associated with Multiple Acyl-CoA Dehydrogenase Deficiency (MADD). However, recent studies have also found that ETFDH plays an important role in the development of several diseases [55, 56], suggesting the need for future research in order to explore its association with biological pathways that drive PTSD.Fms-related receptor tyrosine kinase 3 ligand (FLT3LG) is a growth factor that binds to and forms non-covalent dimers with FLT3 (CD135), acting as an agonist [57]. FLT3LG stimulates the differentiation and proliferation of dendritic cells [58] and has a potential therapeutic value in treating autoimmune diseases and tumors [57, 59]. However, there is relatively limited research on FLT3LG, and further exploration is needed regarding its expression levels, functions, and clinical value in various diseases [60]. Dual-specificity tyrosine phosphorylation regulated kinase 1A (DYRK1A) is an evolutionarily conserved protein kinase and the most extensively studied member of the dual-specificity tyrosine-regulated kinase (DYRK) family. Studies have shown that it is involved in the development of many diseases, and both low and high expression levels of the DYRK1A protein contribute significantly to disease pathology [61]. A recent study suggests that elevated plasma levels of DYRK1A might help prevent neurodegenerative diseases [62], highlighting the potential value of this target in detecting, monitoring, and managing neurodegenerative disorders. As PTSD is a chronic mental disorder, there is a theoretical basis to further explore the role of DYRK1A in neuropsychiatric disorders. Decorin (DCN) is an extracellular matrix protein belonging to the family of small leucine-rich proteoglycans. As a multifunctional molecule, DCN assembles the extracellular matrix and regulates the biological activity of cellular growth factors. Studies [63–67] have shown that DCN acts as a ligand for various cytokines and growth factors by directly or indirectly interacting with signaling molecules involved in cell growth, differentiation, proliferation, adhesion, and migration. The multifunctional nature of DCN makes it a potential therapeutic target for various diseases and shows promising clinical and research applications [68]. FKBP prolyl isomerase 8 (FKBP8) is a member of the FK506-binding protein family, usually found in the mitochondria and the endoplasmic reticulum. FKBP8 plays a critical role in cellular functions, including protein transport and folding [69]. It has been found that FKBP8 plays a vital role in intracellular transport, protein folding, cell apoptosis, cell growth, and differentiation. It also participates in various cellular processes associated with immune regulatory effects [69, 70]. It is worth further investigation into whether FKBP8 plays a role in the occurrence and development of PTSD. At the same time, it should be emphasized that gene expression is generally regulated at transcription and translation levels. The correlation between the expression levels of mRNA and its corresponding protein depends on many regulatory factors and metabolic processes. The transcription process from DNA to mRNA and then to protein involves a set of expression regulation mechanisms that drive mRNA formation and include regulatory activities such as post-transcriptional regulation, epigenetic modifications (such as DNA methylation), and post-translational regulation. The abundance of mRNA and protein expression may not be consistent, and the differences between the two can also suggest more biological significance and regulatory mechanisms. Therefore, a detailed exploration of the specific biological gene expression processes is needed.

One of the limitations of our study is that only two data sets were included in this article. If there is bias in these two data sets, it may affect the results of the analysis. At the same time, the results of potential biomarkers and therapeutic targets screened by bioinformatics can only be used for theoretical guidance to facilitate researchers’ design of future clinical trials. Another limitation is that we did not analyze factors such as race, gender, education level, and dietary habits that may affect outcomes. However, the results of this study can be seen as a supplemental and validating contribution to existing theories. It informs researchers of the potential biological mechanisms and pathways that need to be considered in all areas of PTSD research. This can aid in developing intervention strategies that target these biomarkers as well as the related biological pathways. Our study also has the potential to help expand the pharmacological approaches used to prevent and treat individuals who experience traumatic stress.

## Conclusions

This study utilized a bioinformatic analytic approach to screen potential biomarkers and therapeutic targets (VNN1, SERPINB2, ETFDH, FLT3LG, DYRK1A, DCN, FKBP8) related to PTSD due to TBI. The validation confirmed the potential value of these targets. In addition, by comparing data with other trauma-type patients, it was found that PTSD exhibits different mechanisms under the influence of different trauma characteristics. This provides new insights into the pathophysiology of PTSD as well as valuable references for future clinical practice.

## Data Availability

Publicly available data sets were analyzed in this study. The data sets generated and/or analyzed during the current study are available in the GEO repository [https://www.ncbi.nlm.nih.gov/geo/query/acc.cgi?acc=GSE98793; https://www.ncbi.nlm.nih.gov/geo/query/acc.cgi?acc=GSE164805; https://www.ncbi.nlm.nih.gov/geo/query/acc.cgi?acc=GSE5972].

## Abbreviations

TBI: Traumatic brain injury
PTSD: Post-traumatic stress disorder
GEO: Gene expression omnibus
DEGs: Differentially expressed genes
PPI: Protein–protein interaction
CC: Cellular component
BP: Biological process
MF: Molecular function
